# Extracellular Spermine Activates DNA Methyltransferase 3A and 3B

**DOI:** 10.3390/ijms20051254

**Published:** 2019-03-12

**Authors:** Taro Fukui, Kuniyasu Soda, Koichi Takao, Toshiki Rikiyama

**Affiliations:** 1 Department of Surgery, Saitama Medical Center, Jichi Medical University, Saitama-city, Saitama 330-8503, Japan; d1423@jichi.ac.jp (T.F.); trikiyama@jichi.ac.jp (T.R.); 2 Cardiovascular Research Institute, Saitama Medical Center, Jichi Medical University, Saitama-city, Saitama 330-8503, Japan; 3 Laboratory of Cellular Physiology, Department of Clinical Dietetics & Human Nutrition, Faculty of Pharmaceutical Sciences, Josai University, Sakado, Saitama 350-0295, Japan; ktakao@josai.ac.jp

**Keywords:** gene methylation, methyltransferase, DNA methyltransferase (DNMT), polyamine, spermine, ornithine decarboxylase (ODC), D,L-alpha-difluoromethylornithine (DFMO), adenosylmethionine

## Abstract

We first demonstrated that long-term increased polyamine (spermine, spermidine, putrescine) intake elevated blood spermine levels in mice and humans, and lifelong consumption of polyamine-rich chow inhibited aging-associated increase in aberrant DNA methylation, inhibited aging-associated pathological changes, and extend lifespan of mouse. Because gene methylation status is closely associated with aging-associated conditions and polyamine metabolism is closely associated with regulation of gene methylation, we investigated the effects of extracellular spermine supplementation on substrate concentrations and enzyme activities involved in gene methylation. Jurkat cells and human mammary epithelial cells were cultured with spermine and/or D,L-alpha-difluoromethylornithine (DFMO), an inhibitor of ornithine decarboxylase. Spermine supplementation inhibited enzymatic activities of adenosylmethionine decarboxylase in both cells. The ratio of decarboxylated S-adenosylmethionine to S-adenosyl-L-methionine increased by DFMO and decreased by spermine. In Jurkat cells cultured with DFMO, the protein levels of DNA methyltransferases (DNMTs) 1, 3A and 3B were not changed, however the activity of the three enzymes markedly decreased. The protein levels of these enzymes were not changed by addition of spermine, DNMT 3A and especially 3B were activated. We show that changes in polyamine metabolism dramatically affect substrate concentrations and activities of enzymes involved in gene methylation.

## 1. Introduction

Polyamines are linear aliphatic hydrocarbons with three or more primary amino groups. The representative polyamines are spermidine (three amino groups) and spermine (four amino groups). Putrescine, a precursor of polyamine, has two amines and is therefore referred to as a diamine, and its biological activities differ from those of polyamines [1,2]. For example, polyamines suppress the production of pro-inflammatory cytokines from immune cells upon stimulation with lipopolysaccharide and phorbol 12-myristate 13-acetate [2] and decrease the amount of lymphocyte function-associated antigen 1 (LFA-1) on the cell membrane of immune cells [1], while putrescine seem not to have such biological activities [1,2,3]. Polyamines are synthesized within cells and are essential for functions including differentiation and proliferation. The enzyme activities for polyamine, especially those of ornithine decarboxylase (ODC), synthesis decrease with aging [4,5]. However, the aging-associated decline in polyamine concentrations in tissues and blood cells are not remarkable in healthy adult animals and humans [1,6,7,8], and large inter-individual differences are found [1,7]. Similarly, urinary polyamine excretion, which reflects blood polyamine concentrations, does not change with age during adulthood [8,9]. The age-associated decrease in polyamine concentrations reflect their rapid decline during early life, and no decrease is observed in healthy adult animals and humans.

In addition to *de novo* synthesis, cells can take up polyamine from the extracellular space through a polyamine transporter in the cell membrane. For example, polyamines locally administered in the body [10] and ingested into the intestinal tract are absorbed quickly [11], and are distributed to all organs and tissues [10]. The major sources of polyamines are thought to be foods and synthesis by intestinal microbiota, because suppression of the polyamine supply from both foods and the intestinal microbiota results in decreased blood polyamine concentrations [12,13]. The exact biological mechanisms underlying the large inter-individual differences in blood polyamine concentrations are not known, however, one factor is thought to be differences in the amount of polyamines supplied from the intestinal lumen and in the intestinal environment that are also likely to affect polyamine synthesis. We have shown that a long-term increase in the polyamine supply from food, in which spermidine concentrations are about 2 to 4 times higher than those of spermine, gradually increases blood polyamine levels, especially spermine levels, in humans and mice [14,15]. And, life-long consumption of high-polyamine chow by mice inhibited aging associated pathological changes, and extended lifespan [14,16].

**Figure 1:**
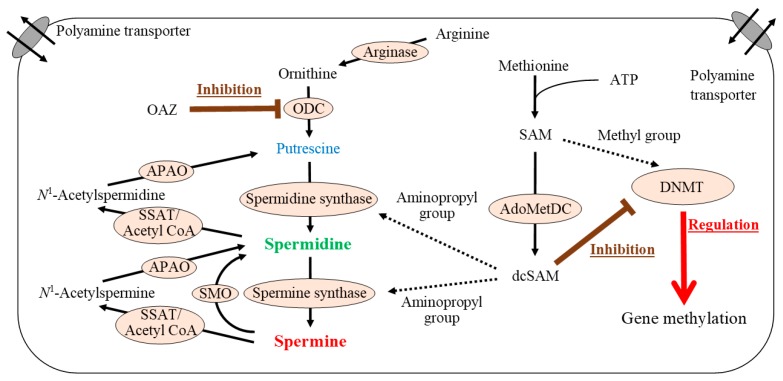
Polyamine synthesis and gene methylation. Ornithine produced from arginine is converted to putrescine by the action of ornithine decarboxylase (ODC), a rate-limiting enzyme in polyamine synthesis. Spermidine is synthesized by addition of an aminopropyl group supplied from decarboxylated S-adenosylmethionine (dcSAM) via the action of spermidine synthase. A second aminopropyl group can be added to spermidine by spermine synthase to produce spermine. When spermine is supplied from extracellular sources as a result of increased polyamine intake, spermidine is produced by the degradation of spermine via spermidine/spermine *N*^1^-acetyltransferase (SSAT)/acetyl coenzyme A (acetyl CoA) and *N*^1^-acetylpolyamine oxidase (APAO). dcSAM is synthesized from SAM by enzymatic activity of S-adenosylmethionine decarboxylase (AdoMetDC). SAM is synthesized from methionine and adenosine, and serves as a methyl-group donor in vivo. dcSAM concentrations have an inverse association with DNMT activity. AdoMetDC: S-adenosylmethionine decarboxylase, APAO: *N*^1^-acetylpolyamine oxidase, ATP: adenosine triphosphate, dcSAM: decarboxylated S-adenosylmethionine, DNMT: DNA methyltransferase, OAZ: ODC antizyme-1, ODC: ornithine decarboxylase, SAM: S-adenosylmethionine, SMO: spermine oxidase, SSAT: spermidine/spermine *N*^1^-acetyltransferase.

There is an overwhelming scientific consensus supporting the important role of epigenetic, especially gene methylation, changes in aging-associated pathologies and lifespan alteration [17,18]. Polyamine metabolism is closely associated with regulation of gene methylation (Figure 1). Polyamines are synthesized from arginine and S-adenosyl-L-methionine (SAM). SAM, produced from adenosine and methionine, is a methyl-group donor. Methylation of genes and proteins such as histones is directly involved in modulation of many biological functions. When methyl groups are added to DNA cytosines in gene promoter regions as an epigenetic modification, transcription of the corresponding gene is suppressed, and when methyl groups are removed from the promoter region, transcription is increased. DNA methyltransferases (DNMTs) are enzymes that catalyze transfer of a methyl group from SAM to a cytosine. The concentration of decarboxylated S-adenosylmethionine (dcSAM), which is produced from SAM by the enzymatic activity of S-adenosylmethionine decarboxylase (AdoMetDC), and also the dcSAM to SAM ratio, are closely associated with DNMT activity [19].

**Figure 2:**
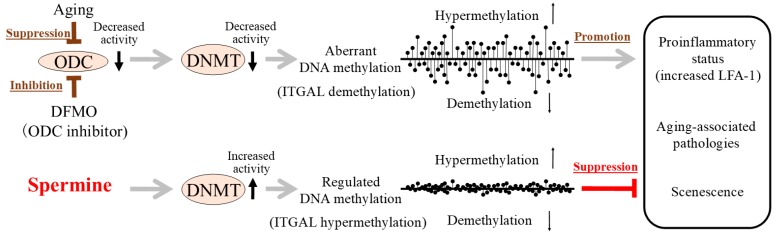
Aging, ODC activity, DNA methyltransferase (DNMT) activity, gene methylation status, and progression of aging associated pathologies and senescence (summary of the results of our previous studies).(Upper) Aging is associated with decreases in ODC and DNMT activities, increased aberrant methylation status (increased demethylation in certain areas and increased hypermethylation in other areas) of entire genome, and enhanced pro-inflammatory status. D,L-alpha-difluoromethylornithine (DFMO)-induced ODC inhibition caused decreased DNMT activities, increased aberrant methylation, increased demethylation of the LFA-1 promoter area (ITGAL), and enhanced pro-inflammatory status (increased LFA-1 protein). (Lower) Spermine supplementation reversed changes induced by the inhibition of ODC by DFMO. Increased polyamine intake elevated blood spermine levels in mice and humans, and lifelong intake of high-polyamine chow inhibited aging-associated increase in aberrant DNA methylation and LFA-1 expression, inhibited aging-associated pathologies, and extended lifespan of mice. DFMO: D,L-alpha-difluoromethylornithine, ODC: ornithine decarboxylase, DNMT: DNA methyltransferase, ITGAL: promoter area of LFA-1, LFA-1: lymphocyte function-associated antigen 1.

Aging is associated with enhanced demethylation of DNA in various organs and tissues in several animals and humans [20,21]. However, increased hypermethylation associated with age has also been reported in some genes [22,23]. The aging-associated changes in aberrant DNA methylation status, namely increased de-methylation in some areas and hypermethylation in other areas, are considered to be among the most important mechanisms underlying aging-associated pathologies. Our previous studies showed that D,L-alpha-difluoromethylornithine (DFMO)-induced ODC inhibition caused aberrant methylation in Jurkat cells, while spermine supplementation reversed this condition [16,24]. And, the site responsible for LFA-1 expression was demethylated and associated with increased LFA-1 protein levels after ODC inhibition, and spermine supplementation reversed the demethylation and increase of LFA-1 protein levels (Figure 2). Aging is associated with decreases in ODC [5] and DNMT activities [25], increased aberrant methylation status of entire genome, and enhanced demethylation of the LFA-1 promoter area in association with increases in LFA-1 protein levels [1,16,26]. In a murine model involving chows with different polyamine concentrations, the methylation status of the entire genome in old mice fed regular chow showed an increase in aberrant methylation of entire genome in association with increases in LFA-1 protein levels. However, lifelong intake of high-polyamine chow prepared by adding synthetic polyamines prevented aging-associated increases in aberrant methylation and LFA-1 expression (Figure 2) [14,16].

Changes in polyamine metabolism influencing SAM and dcSAM concentrations and AdoMetDC activity may affect DNMT activities and gene methylation. However, how changes in polyamine metabolism affect substrate concentrations and enzymatic activities involved in gene methylation is not known in detail. In this study, we investigated the effects of decreased polyamine synthesis and of extracellular polyamine supply on substrate concentrations and enzyme activities involved in polyamine metabolism. In our previous studies, spermine concentrations in blood cells of humans and mice fed a high-polyamine diet increased by 1.1- to 1.5-fold, whereas there was no significant increase in spermidine concentration [15]. And, 500 µM spermine in culture supernatant of human peripheral blood mononuclear cells increased intracellular spermine concentrations similar to those in vivo studies [1]. Spermidine at this concentration showed similar bioactivities (e.g., suppression of pro-inflammatory cytokine synthesis and LFA-1 expression) [1,2], but this intracellular spermidine concentration markedly exceeds (by 3 to 4 times) the physiological range [1]. Therefore, in this study, we used spermine to replicate physiological effects in vivo.

## 2. Results

### 2.1. Determination of Culture Condition

Polyamines are contained in all cells in high micromolar to low millimolar quantities. Our previous study showed that spermine concentrations of up to 1 mM for up to 80 h were not toxic [1]. And, the previous studies have established optimal concentrations for DFMO of 3.0 mM in Jurkat cells [24]. 

**Figure A1:**
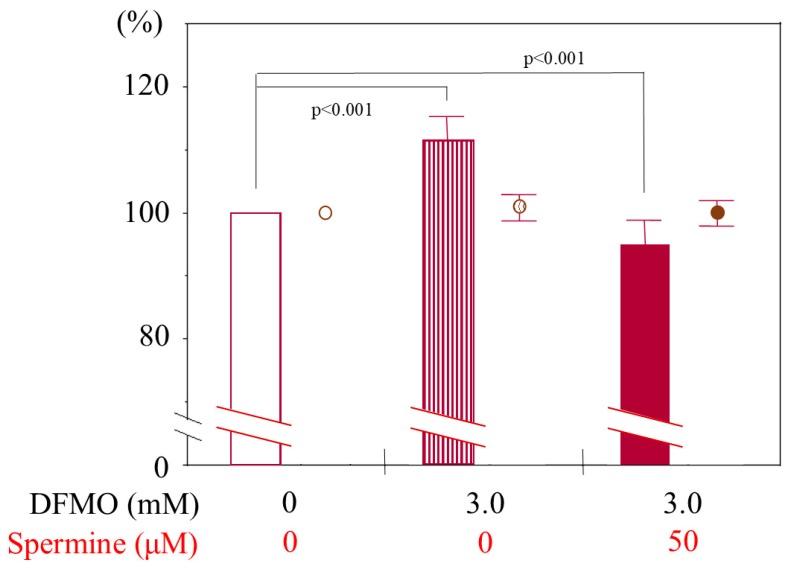
Changes in LFA-1 expression and cell viability of Jurkat cells cultured with DFMO and spermine. Expressions of CD11a and Viaprobe on Jurkat cells cultured for 72 h in various conditions were analyzed by flow cytometry. The values were relative to the control (cells cultured in unsupplemented culture medium). Data are shown as means and standard deviations. Bars indicate percentage of mean fluorescent intensities of twelve samples (*n* = 12) in each culture condition. Circles indicate percentage of viable cells (*n* = 3). DFMO (3mM) and spermine (500 µM) did not change cell viability. 3mM of DFMO for 3 days increased mean fluorescent intensity of CD11a, and spermine (500µM) with DFMO decreased LFA-1 expression without affecting cell viability. DFMO: D,L-alphadifluoromethylornithine-hydrochloride.

Flow cytometric examination revealed that DFMO treatment (3 mM) for 3 days increased mean fluorescent intensity (MFI) of CD11a. Spermine treatment alone and DFMO plus spermine treatment decreased the MFI of CD11a staining. The percentage of cells negative for ViaProbe was not changed, indicating that these treatments decreased LFA-1 expression without affecting cell viability (Figure A1).

**Figure A2:**
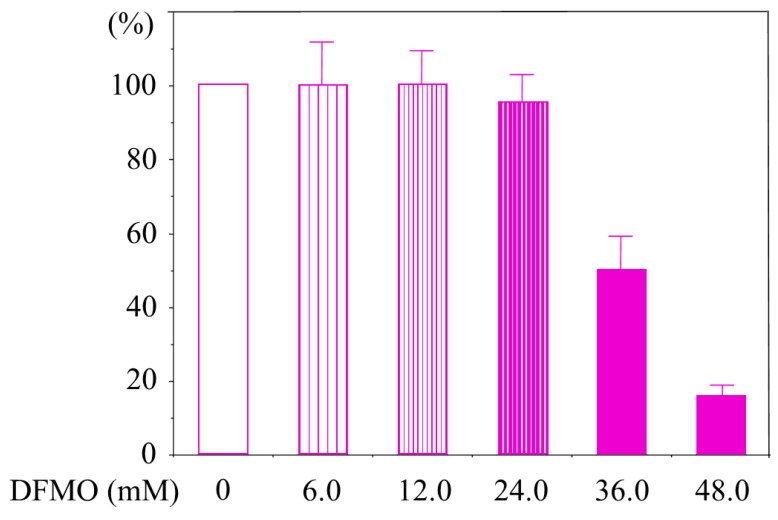
Cell viability and activity analyses (HMEpCs). Viability of HMEpCs cultured in 96-well with various concentrations of DFMO was analyzed by MTT assay. DFMO concentrations of up to 24 mM were not toxic to HMEpCs. The values were relative to the control (cells cultured in unsupplemented culture medium). Data are shown as means and standard deviations of four experiments (*n* = 4). HMEpCs: human mammary epithelial cells, MTT: 3-(4,5-Dimethyl-2-thiazolyl)-2,5-diphenyl-2H-tetrazolium bromide.

HMEpCs does not express LFA-1, therefore we determined the maximum concentration of DFMO that does not decrease cell viability and cell activity. 3-(4,5-dimethylthiazol-2-yl)-2,5-diphenyltetrazolium bromide (MTT) assay revealed that DFMO concentrations of up to 24 mM were not toxic to HMEpCs (Figure A2).

### 2.2. DFMO and Spermine-Induced Changes in Intracellular Polyamine Concentrations

**Figure 3:**
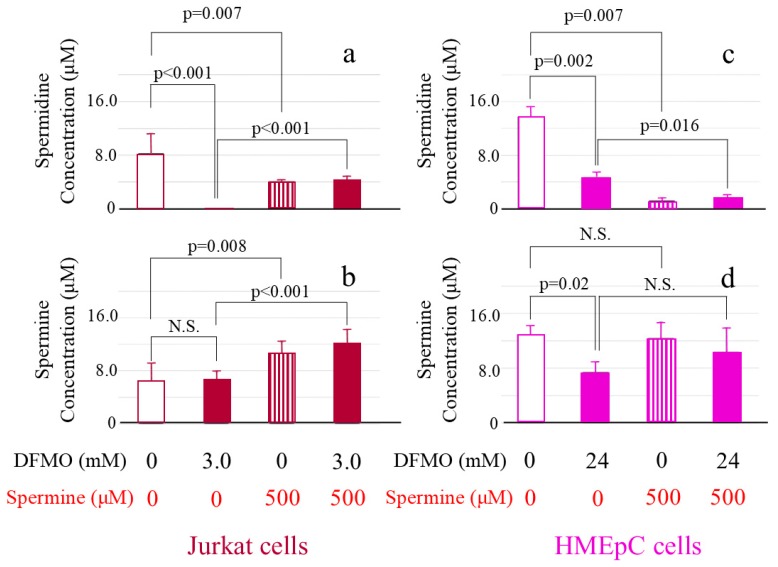
Changes in intracellular polyamine concentrations in cells cultured with D,L-alpha-difluoromethylornithine (DFMO) and spermine. Cells cultured for 72 h in different conditions were collected and intracellular polyamine concentrations were determined by reversed-phase high-performance liquid chromatography (HPLC). (**a**): Intracellular spermidine concentrations in Jurkat cells. (**b**): Intracellular spermine concentrations in Jurkat cells. (**c**): Intracellular spermidine concentrations in human mammary epithelial cells (HMEpCs). (**d**): Intracellular spermine concentrations in HMEpCs. Data are shown as means and standard deviations of six samples (*n* = 6) for each culture condition. N.S.: not significant.

We established optimal concentrations for DFMO of 3.0 mM in Jurkat cells and 24 mM in human mammary epithelial cells (HMEpCs) and for spermine of 500 µM in both cell types. The intracellular spermidine and spermine concentrations in cultured Jurkat cells (controls = cells cultured in unsupplemented culture medium) were 8.27 ± 3.01 µM/1.0 × 10^6^ cells and 6.45 ± 2.60 µM/1.0 × 10^6^ cells, respectively. When Jurkat cells were cultured with 500 µM spermine, intracellular spermine concentration significantly increased to 10.63 ± 1.65 μM/1.0 × 10^5^ cells (*p* = 0.008) (Figure 3b), whereas the spermidine concentration of 4.09 ± 0.28 μM/1.0 × 10^5^ cells showed significant decrease (Figure 3a). In Jurkat cells cultured with 3 mM DFMO, spermidine decreased to below the detection limit (*p* < 0.001) (Figure 3a), whereas the spermine concentration was 6.76 ± 1.03 µM/1.0 × 10^6^ cells, showing no significant decrease (*p* = 0.793) (indicated as N.S. in Figure 3b). After addition of 500 µM spermine to cells cultured with DFMO, both spermidine (4.35 ± 0.61 μM/1.0 × 10^5^ cells) (Figure 3a) and spermine (12.20 ± 1.97 μM/1.0 × 10^5^ cells) (Figure 3b) concentrations increased significantly (*p* < 0.001). When spermine and spermindine concentrations were compared between cells cultured with spermine and those cultured with DFMO and spermine, there were no differences in intracellular spermidine (*p* = 0.365) and spermine (*p* = 0.184) concentrations.

The intracellular spermidine and spermine concentrations in control HMEpCs were 13.79 ± 1.41 µM/1.0 × 10^5^ cells and 12.93 ± 1.30 µM/1.0 × 10^5^ cells, respectively. When cells were cultured with 500 µM spermine, spermidine significantly decreased to 1.22 ± 0.39 μM/1.0 × 10^5^ cells (*p* = 0.007) (Figure 3c), whereas the spermine concentration of 12.39 ± 2.37 μM/1.0 × 10^5^ cells showed no significant difference from that in control cells (Figure 3d). Intracellular spermidine in cells cultured with 24 mM DFMO decreased significantly to 4.69 ± 0.77 μM/1.0×10^5^ cells (*p* = 0.002) (Figure 3c) and spermine also decreased to 7.42 ± 1.43 μM/1.0 × 10^5^ cells (*p* = 0.02) (Figure 3d). After adding 500 µM spermine to cells cultured with DFMO, the concentration of spermidine decreased to 1.62 ± 0.48 μM/1.0 × 10^5^ cells (*p* = 0.016) (Figure 3c) and that of spermine slightly increased (10.40 ± 3.53 μM/1.0 × 10^5^ cells), but the change was not statistically significant (Figure 3d). When intracellular spermidine and spermine concentrations were compared between cells cultured with spermine and those cultured with DFMO and spermine, there were no differences (spermidine; *p* = 0.459, spermine; *p* = 0.948).

### 2.3. AdoMetDC Activity, SAM Concentration, dcSAM Concentration, and dcSAM/SAM Ratio

**Figure 4:**
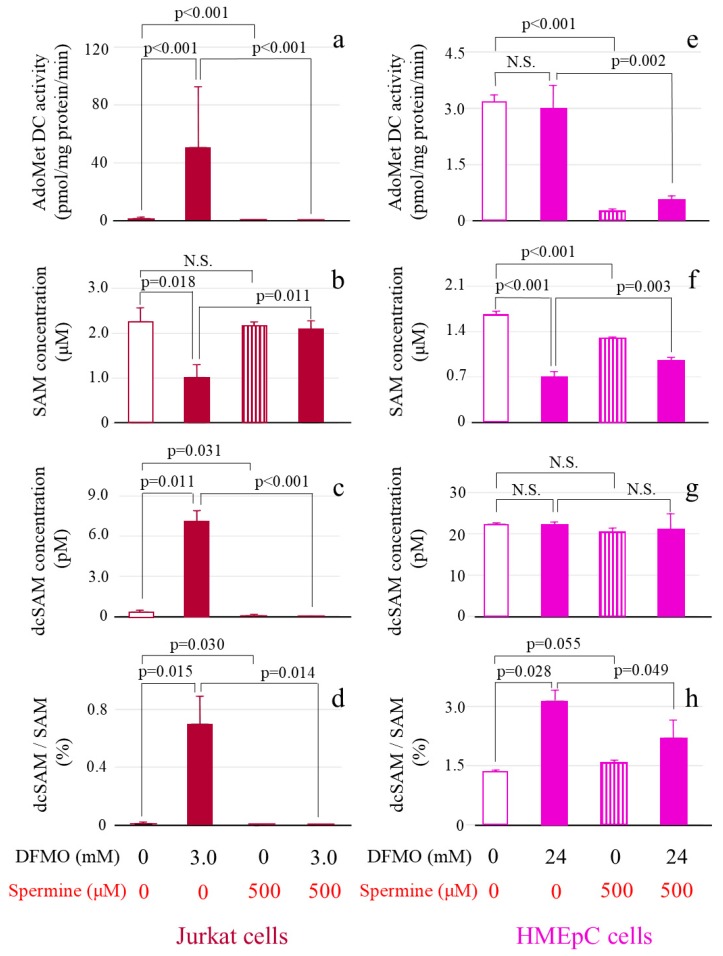
The effect of DFMO and spermine on AdoMetDC activity, SAM concentration, dcSAM concentration, and dcSAM/SAM ratio. AdoMetDC activity: Cells cultured for 72 h in different conditions were collected and the ^14^CO_2_ released when ^14^C-labeled adenosyl-L-methionine (carboxyl-^14^C) was converted to dcSAM by AdoMetDC was measured using a scintillation counter. SAM and dcSAM concentrations in Jurkat cells (1.0 × 10^6^) and HMEpCs (1.0 × 10^5^) were measured by HPLC. (**a**): AdoMetDC activity (pmol/mg protein/min) in Jurkat cells. (**b**): SAM concentration (μM) in Jurkat cells. (**c**): dcSAM concentration (pM) in Jurkat cells. (**d**): dcSAM/SAM ratio (%) in Jurkat cells. (**e**): AdoMetDC activity (pmol/mg protein/min) in HMEpCs. (**f**): SAM concentration (μM) in HMEpCs. (**g**): dcSAM concentration (pM) in HMEpCs. (**h**): dcSAM/SAM ratio (%) in HMEpCs. Data are shown as means and standard deviations of six samples (*n* = 6) for each culture condition. N.S.: not significant.

AdoMetDC activity in Jurkat cells (50.81 ± 41.52 pmol/mg protein/min) cultured with DFMO markedly increased in comparison with that in control cells (i.e., cultured without DFMO or spermine) (1.56 ± 0.77 pmol/mg protein/min) (*p* < 0.001) (Figure 4a). When cells treated with DFMO were cultured simultaneously with spermine, this increase in AdoMetDC was significantly inhibited (0.008 ± 0.18 pmol/mg protein/min) (*p* < 0.001) (Figure 4a). With addition of spermine alone, AdoMetDC activity (0.034 ± 0.18 pmol/mg protein/min) was lower than that in control cells (1.56 ± 0.77 pmol/mg protein/min) (*p* < 0.001) (Figure 4a).

The SAM concentration in Jurkat cells cultured with DFMO (1.02 ± 0.28 μM) was lower than that in control cells (2.27 ± 0.30 μM) (*p* = 0.018) (Figure 4b). No change in SAM concentration occurred after spermine addition alone (2.16 ± 0.08 μM) (*p* = 0.40 vs. control culture), but there was an increase in SAM in cells cultured with DFMO and spermine (2.10 ± 0.19 μM) (*p* = 0.011 vs. DFMO culture (1.02 ± 0.28 μM) (Figure 4b).

The dcSAM concentration significantly increased in Jurkat cells cultured with DFMO (7.12 ± 0.74 pM) (*p* = 0.001 vs. control culture (0.34 ± 0.12 pM) and significantly decreased in response to spermine addition in the absence (0.043 ± 0.074 pM) (*p* = 0.031 vs. control culture (0.34 ± 0.12 pM) or presence (dcSAM concentration below the detection limit) (*p* < 0.001 vs. DFMO culture (7.12 ± 0.74 pM) of DFMO (Figure 4c). 

The dcSAM/SAM ratio in Jurkat cells was low in control cells (0.015 ± 0.006%) and markedly increased in cells cultured with DFMO (0.70 ± 0.19%) (*p* = 0.015 vs. control culture) (Figure 4d). This ratio decreased in cells cultured with spermine in the absence of DFMO (0.002 ± 0.003%) (*p* = 0.03 vs. control culture (0.015 ± 0.006%) and the presence of DFMO (0.70 ± 0.19%) (*p* = 0.014 vs. DFMO culture (below the detection limit) (Figure 4d).

When HMEpCs were cultured with DFMO (AdoMetDC activity 2.99 ± 0.47 pmol/mg protein/min), the increase in AdoMetDC activity observed in Jurkat cells did not occur (*p* = 0.59 vs. control (3.17 ± 0.14 pmol/mg protein/min) (Figure 4e). However, AdoMetDC activity markedly decreased in spermine-treated cells in the absence (0.28 ± 0.045 pmol/mg protein/min) (*p* < 0.001 vs. control culture) and presence of DFMO (0.57 ± 0.13 nmol/mg protein/min) (*p* = 0.002 vs. DFMO culture) (Figure 4e).

In HMEpCs cultured with DFMO, the SAM concentration decreased (0.71 ± 0.074 μM) (*p* < 0.001 vs. control culture (1.67 ± 0.040 μM) (Figure 4f), but the dcSAM concentration did not change (control 22.44 ± 0.023 pM vs. culture with DFMO 22.27 ± 0.45 pM) (Figure 4g). SAM was decreased by addition of spermine (1.30 ± 0.009 μM) (*p* < 0.001 vs. control culture (1.67 ± 0.040 μM) (Figure 4f) but increased by adding spermine to cells cultured with DFMO (0.96 ± 0.035 μM) (*p* < 0.003 vs. DFMO culture (0.71 ± 0.074 μM) (Figure 4f). No differences in the dcSAM concentration were found among culture conditions (Figure 4g). 

The dcSAM/SAM ratio in HMEpCs was increased in cells cultured with DFMO (3.15 ± 0.27%) (*p* = 0.028 vs. control culture (1.35 ± 0.031%), and its increase was suppressed by adding spermine to cells cultured with DFMO (2.11 ± 0.04%) (*p* = 0.049 vs. DFMO culture (3.15 ± 0.27%) (Figure 4h).

### 2.4. DNMT Levels and Activities

Tumor cells such as Jurkat cells grow rapidly, however multiplication of normal cells such as HMEpCs was very slow. Therefore, it was difficult to secure enough HMEpCs to measure protein levels and DNMT activities. Thus Jurkat cells were cultured for examination of protein levels and activities of DNMTs because sufficient enzyme can be obtained from these cells for measurement. 

**Figure 5:**
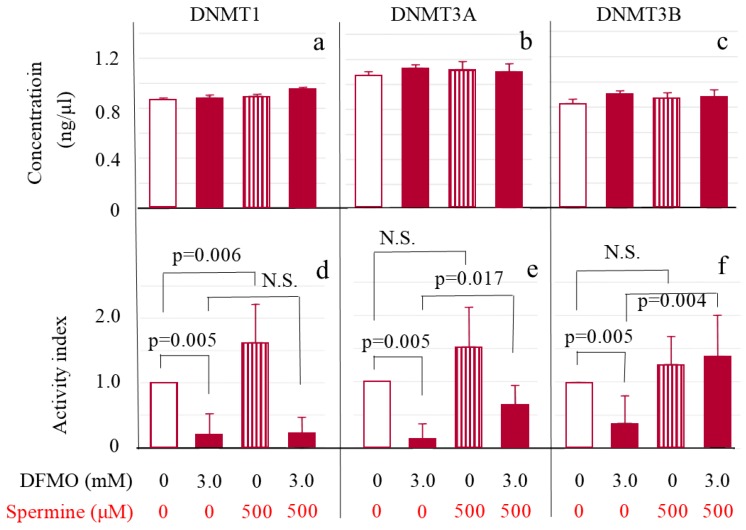
ODC inhibition suppressed DNMT 1, 3a, and 3b activities and spermine supplementation activated DNMT 3a and 3b without affecting their protein levels in Jurkat cells. Upper Figure: amount of DNMT subtypes. Nuclear proteins were extracted using 1.0 μL of extraction buffer per 1.0 × 10^6^ Jurkat cells and each DNMT enzyme concentration in extracts was determined. (**a**): DNMT 1; (**b**): DNMT 3A; (**c**): DNMT 3b. Lower Figure: Enzyme activity per amount of each DNMT subtype. Activity was determined after adjustment to make DNMT protein concentrations equivalent. DNMT activities are shown using indexes that were calculated by dividing the measured activity by that of the control culture for each subtype. (**d**): DNMT 1; (**e**): DNMT 3A; (**f**): DNMT 3b. Data are shown as means and standard deviations of six samples (*n* = 6) in each culture condition. N.S.: not significant.

DNMT 1 (Figure 5a), 3A (Figure 5b) and 3B (Figure 5c) protein levels in Jurkat cells were not influenced either by DFMO or spermine. For measurement of enzymatic activities of three DNMTs, nuclear protein solutions were adjusted with assay buffer to make the protein concentrations of all DNMTs equivalent. The adjusted enzyme activities for DNMT 1, 3A and 3B all markedly decreased in cells cultured with DFMO in comparison with control cells (DNMT 1 0.19 ± 0.33 vs. control cells 1.00 ± 0.00 (Figure 5d); DNMT 3A 0.14 ± 0.22 vs. control cells 1.00 ± 0.00 (Figure 5e); and DNMT 3B 0.39 ± 0.41 vs. control cells 1.00 ± 0.00 (Figure 5f)) (*p* = 0.005 for each DNMT subtype). Addition of spermine significantly activated DNMT 1 (1.63 ± 0.60, *p* = 0.006 vs. control culture) (Figure 5d) and tended to activate DNMT 3A (1.54 ± 0.59) (Figure 5e) and 3B (1.27 ± 0.41) (Figure 5f) with no statistical significance (*p* = 0.069 for each). Addition of spermine to cells cultured with DFMO did not change DNMT 1 activity (0.23 ± 0.24) (Figure 5d), but DNMT 3A (1.54 ± 0.59, *p* = 0.017) (Figure 5e) and DNMT 3B (1.40 ± 0.60, *p* = 0.004) (Figure 5f) were activated when compared to cells cultured with DFMO.

## 3. Discussion

In this study, we have shown that changes in polyamine metabolism affect DNMT activities. Inhibitory effects of DFMO on polyamine concentrations and on activation of AdoMetDC activity varied between Jurkat cells and normal HMEpCs, which may be due to the difference of capability to maintain polyamine homeostasis. Changes in spermine concentration upon spermine supplementation also varied between the two cell types. However, changes in intracellular spermidine concentration were found in both cell types cultured with spermine, which shows that intracellular polyamine metabolism was influenced by the extracellular spermine supply. In Jurkat cells treated with DFMO, spermine supplementation increased both spermine and spermidine concentrations. Since spermidine levels were under detectable levels in cells co-cultured with 3 mM of DFMO, an increase in spermidine concentration by spermine supplementation in DFMO-treated Jurkat cells reflects increased spermine catabolism. Spermine can be converted to spermidine by the enzymatic activities of SSAT/Acetyl CoA and APAO. Spermine supplementation to control Jurkat cells (cultured in unsupplemented culture medium) decreased spermidine concentrations, suggesting ODC inhibition by a negative feedback mechanism. Decreases in spermidine concentrations by spermine supplementation were observed in HMEpCs whether cells were co-cultured with DFMO or not, suggesting that 24 mM of DFMO could not completely inhibit ODC activity. Furthermore, AdoMetDC activity was markedly decreased by spermine in both cells, suggesting that intake of spermine into cells induces a negative feedback mechanism to inhibit polyamine synthesis.

As observed in both Jurkat cells and HMEpCs, dcSAM concentration in vivo is generally a few percent of the SAM concentration [27]. The DFMO-induced changes in AdoMetDC activity and SAM and dcSAM concentrations observed in Jurkat cells were similar to the results of previous studies [28,29,30]. DFMO significantly increased AdoMetDC activity and dcSAM concentration, whereas SAM concentration decreased in Jurkat cells. Although AdoMetDC activity in HMEpCs did not change significantly on treatment with DFMO, the effect of spermine supplementation was similar to that observed in Jurkat cells and in a previous report in which spermine inhibited DFMO-induced increases in AdoMetDC activities in Ehrlich ascites-carcinoma cells [30]. 

dcSAM is likely to have inhibited activation of DNMT through competition with SAM [31,32]. Thus, in Jurkat cells, dynamic changes in dcSAM concentration occurred with DFMO and spermine, with a negative relationship between the dcSAM concentration and DNMT activity, especially of DNMT 3B. Changes in dcSAM concentration were not significant in HMEpCs, however, spermine suppressed DFMO-induced increases in dcSAM/SAM ratio. An inverse relationship between the dcSAM/SAM ratio and DNMT activity was reported previously [33]. AdoMetDC activities and the ratio of dcSAM to SAM were relatively similar among the three conditions tested (DFMO(-)spermine(-), DFMO(-)spermine(+), DFMO(+)spermine(+)) on Jurkat cells, though not in cells co-cultured with DFMO alone. However, the changes in DNMT activities were not necessarily the same among these three conditions. These findings indicate that some factor(s) other than the dcSAM concentration and dcSAM/SAM ratio may affect DNMT activities and gene methylation [34].

One of the very interesting findings in this study is that changes in polyamine metabolism influenced DNMT activities without affecting their protein levels. The effect of decreased ODC activity was examined previously, and it was reported that protein levels of DNMT 3B in human oral cancer cells decreased when the ODC antizyme-1 gene, which degrades ODC and inhibits its activity, was transfected [35]. The different effects on DNMTs between studies may be due to the different cell lines employed or methods used to inhibit ODC activity. In the present study, the activities of all DNMTs decreased significantly on DFMO treatment. In contrast, spermine activated DNMT 1 when cells were not treated with DFMO. Although such an effect was not observed for DNMT 3A and 3B when spermine alone was added to the culture supernatants, spermine markedly activated DNMT 3B and DNMT 3A when cells were treated with DFMO. Methylation patterns in genomes are considered to be stably inherited by cells in the next generation [36]; however, reversibly modified regions of methylation have also been found [35,37]. DNMT 1 mainly acts to maintain methylation in DNA replication, whereas DNMT 3A and DNMT 3B have important roles in *de novo* methylation [35]. A study showed that the role of DNMT 1 is gradually compensated, at least partially, by DNMT3 [38], suggesting the importance of spermine supplementation for the maintenance of gene methylation status. 

**Figure 6:**
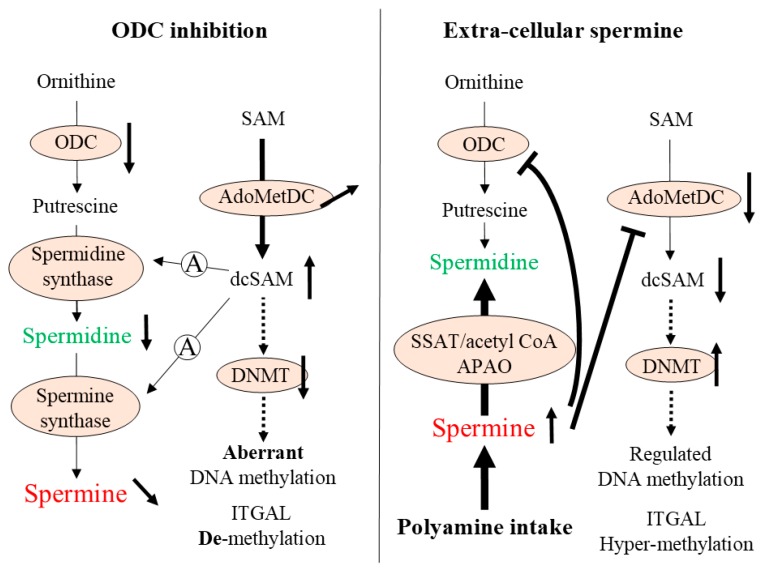
Summary of the study. (Left) When ODC is suppressed, AdoMetDC activity is either activated or unchanged. Decreased ODC results in decreased supply of putrescine for polyamine synthesis. The decreased supply of putrescine does not require an aminopropyl group (A in circle) from dcSAM. Therefore, surplus dcSAM inhibits DNMT 1, 3a, and 3b activities, resulting in aberrant methylation of the entire genome and demethylation of ITGAL. (Right) When spermine is supplied from extracellular sources as a result of increased polyamine intake, intracellular polyamine synthesis is suppressed. Spermidine is produced by the degradation of spermine via SSAT/Acetyl CoA and APAO, and therefore, no aminopropyl group (A in circle) is required for polyamine synthesis. This may result in strong inhibition of AdoMetDC (indicated by thick T-bar), resulting in a decrease of dcSAM concentration or dcSAM/SAM ratio. Decreased dcSAM re-activates DNMT 3a and 3b, resulting in regulation of the methylation status of the entire genome as well as hypermethylation of ITGAL. Hypermethylation of ITGAL suppresses aging-associated enhancement of the pro-inflammatory status by decreasing protein levels of LFA-1. Inhibition of aberrant DNA methylation may result in inhibition of various aging-associated pathological changes. Arrows indicate the metabolic pathway or flow of substances. T-bars indicate inhibitory activity. Dashed arrows indicate the consequence (downstream) induced by the change (upstream). ODC: Ornithine decarboxylase, SAM: S-adenosylmethionine, AdoMetDC: Adenosylmethionine decarboxylase, dcSAM: Decarboxylated S-adenosylmethionine, DNMT: DNA methyltransferase, SSAT: Spermidine/spermine *N*^1^-acetyltransferase; APAO: *N*^1^-acetylpolyamine oxidase, A in circle: Aminopropyl group.

SAM serves as a methyl group donor, therefore increases in SAM concentrations by spermine supplementation indicate increased availability of methyl group. Increased availability of methyl group and increased DNMT activity seem to be the key to maintain DNA methylation status. Supplementation of methyl group by either methionine or SAM affects the DNA methylation status [39,40,41], and decrease DNMT is associated with alteration of methylation status of the entire genome [35,42]. Generally, decreases in ODC activity with aging [4,5,33] is associated with decreases in DNMT activities [25,43] and changes in DNA methylation status [43,44,45]. Aging associated change in DNA methylation status seems to be a non-directional change as it involves both hypermethylation and hypomethylation events [46,47,48]. Alteration of methylation status with aging changes chromatin accessibility, resulting in aberrant gene transcription as well as genomic instability. These factors may be key regulators of the aging process and contributors to the development of aging-associated diseases [49,50,51], including neoplastic growth [52,53,54] and aging itself [55,56,57]. In the previous studies, we have shown that aberrant methylation status induced by inhibiting ODC was almost reversed by spermine supplementation [24], and that life-long consumption of high-polyamine chow by mice inhibited aging-associated changes in methylation status of the entire genome, inhibited aging associated pathological changes, and extended lifespan [14,16]. In addition, increased polyamine intake followed by repeated weak carcinogenic stimuli decreased tumorigenesis in animals [16,58]. The increases in spermine concentrations by continuously increased polyamine intake may compensate for aging-associated decreases in DNMT activities by decreasing dcSAM, and increased SAM availability and increased DNMT activity may attenuate progression of aberrant gene methylation (Figure 6).

## 4. Materials and Methods

### 4.1. Cells and Culture Conditions

Jurkat cells (Human Science Research Resource Cell Bank, Tokyo, Japan) and human mammary epithelial cells (HMEpCs) (Cell Applications, San Diego, CA, USA) were used in this study. Jurkat cells were adjusted to a cell density of 1.0 × 10^6^ cells/mL and incubated in RPMI-1640 culture medium (Sigma-Aldrich, St. Louis, MO, USA) containing 10% human inactivated serum (Cosmo Bio Co., Tokyo, Japan) for 72 h before use for subsequent experiments. HMEpCs were adjusted to a cell density of 5.0 × 10^4^cells/mL, incubated in serum-free medium (MammaryLife™ Comp Kit, Kurabo Industries, Tokyo), and subcultured according to the manufacturer’s protocol. Cells were collected for use in experiments after sufficient passages resulted in a large enough number of cells, and then incubated for 72 h in the following conditions: 1. control culture (Jurkat: RPMI-1640 + 10% human inactivated serum; HMEpCs: serum-free medium); 2. culture mixed with D,L-alpha-difluoromethylornithine (DFMO) (Amine Pharma Institute, Chiba, Japan), an irreversible inhibitor of ODC; 3. culture with spermine (Sigma-Aldrich Japan, Tokyo); and 4. culture with DFMO and spermine. In Jurkat cells, 3.0 mM DFMO and 500 µM spermine were used in accordance with the protocol of a previous study [46]. HMEpCs were cultured with DFMO at different concentrations and examined by 3-(4,5-dimethylthiazol-2-yl)-2,5-diphenyltetrazolium bromide (MTT) assay (In Vitro Toxicology Assay Kit, Sigma-Aldrich Japan) and the highest non-cytotoxic concentrations were determined. This resulted in the use of 24 mM DFMO in subsequent experiments.

### 4.2. Flow Cytometric Analysis and cell Viability Assay

Jurkat cells cultured in various conditions were fixed in 2% paraformaldehyde for 10 min at 4 °C. To cells suspended in phosphate buffered salts (PBS) containing 0.1% bovine serum albumin (BSA), the following antibodies were added (5 mL per 56105 cells): fluorescein isothiocyanate (FITC)-conjugated anti-human CD11a and FITC-ViaProbe (BD Pharmingen, San Jose, California (CA), USA). After incubating for 20 min at 4 °C, cells were washed with 3 times PBS. A FACScan flow cytometer (FACS Calibur, Becton, Dickinson and Company, Franklin Lakes, New Jersey (NJ), USA) with CellQuest analysis software was used to identify 1 × 10^6^ Jurkat cells gated in the lymphocyte light scatter region, which were then further analyzed.

HMEpCs cultured in 96-well with various concentrations of DFMO were incubated for 3–4 h in the presence of 0.35 mg/mL 3-(4,5-Dimethyl-2-thiazolyl)-2,5-diphenyl-2*H*-tetrazolium bromide (MTT) (Sigma-Aldrich, Saint Louis, USA). After color changed, the supernatant was removed, and 100 µl of isopropyl alcohol containing 12 M HCl was added to the wells. The absorption at 570 nm and 690 nm was determined with an automated enzyme immunoassay analyzer (DTX 880 Multimode Detector, Beckman Coulter Inc., Brea, California, USA).

### 4.3. Measurement of Intracellular Polyamine Concentrations

Polyamines were extracted from cells according the previous studies with slight modifications [59]. Cultured cells were centrifuged (Jurkat cells: 250 × *g*, 3 min, 4 °C; HMEpCs: 220 × *g*, 5 min, 4 °C) and precipitates were suspended in 0.2 M perchlorate in 50 µL per 1.0 × 10^6^ Jurkat cells and 1.0 × 10^5^ HMEpCs. The suspended samples were then homogenized (150 W, 10 s, ×3 cycles) using an ultrasonic homogenizer (Ultrasonic Cleaner VS-150, AS One Corporation. (Iuchi Koki Inc.), Nishi-ku, Osaka, Japan) and vortexed. The supernatant (50 µL) obtained by centrifugation (14,000 × *g*, 10 min, 4 °C) was fluorescence-derivatized by reaction with 300 µL of dansyl chloride (10 mg/mL). The dansylated samples were redissolved in acetonitrile (500 µL). The concentrations of polyamines in extracts was determined by reversed-phase high-performance liquid chromatography (HPLC; LC-20AB, Shimadzu Corp., Nakagyo-ku, Kyoto, Japan) using a Capcell pak C18 MG column (Shiseido Co., Chuo-ku, Tokyo, Japan). 10 µL of the dansylated sample was injected per assay. The HPLC conditions were as follows: column oven at 50 °C; flow rate, 0.9 mL/min; two solvent linear gradient, solvent A consisted of 55% (v/v) 10 mM ammonium phosphate (pH 4.4) + 45% (*v*/*v*) acetonitrile, solvent B as 100% acetonitrile; the gradient condition, the solvent B 18% to 100% for 15 min and 100% for further 7 min. The measurement was conducted at an excitation wavelength of 360 nm and an emission wavelength of 500 nm and data were processed using LC Workstation chromatography software (Shimadzu Corp., Kyoto, Japan).

### 4.4. Measurement of AdoMetDC Activity

Jurkat cells cultured in each condition were centrifuged (250 × *g*, 4 °C, 3 min) after washing with 1 × PBS(-). Cell pellets obtained were suspended in 25 mM Tris-HCl buffer (pH 7.2, 300 μL) containing extraction reagents (1 mM dithiothreitol, 1.0 mM EDTA and 0.01% Tween 80), homogenized (150 W, 10 s, × 3), and centrifuged (13,964 × *g*, 4 °C, 30 min) to obtain supernatants containing intracellular proteins.

HMEpCs attached to a culture dish were washed with 1× PBS(-), collected using a cell scraper, homogenized (150 W, 10 s, ×3), and centrifuged (13,964 × *g*, 4 °C, 30 min) after injecting the extraction buffer (300 μL) to obtain supernatants containing intracellular proteins. Protein contents in supernatants were measured by the Bradford method using bovine serum albumin as a standard. 

The measuring principle for AdoMetDC activity is to determine CO_2_ emitted when SAM (carboxyl-^14^C) is converted to dcSAM by AdoMetDC. A filter paper was attached inside a 2 mL Eppendorf tube cap; 20 µL of 250 mM phosphate buffer (pH 7.5) and 15 mM DTT, 15 µL 25 mM putrescine dihydrochloride, and a sample (intracellular protein solution) of 80 µL were mixed; and 10 µL of a SAM cocktail of 4.8 mM cold (i.e., non-radioactive) SAM (New England Biolabs, MA, USA) and 0.2 mM SAM (carboxyl-^14^C) (0.1 µCi/reaction) (American Radiolabeled Chemicals, St. Louis, MO, USA) were added. To adsorb CO_2_ with alkali, 10% KOH was permeated in the filter paper on the cap. The cap was immediately shut to allow production of CO_2_ at 37 °C for 30 min and then the tube was cooled on ice for 15 min. To release dissolved CO_2_, 50 µL 6 M HCl was added to the resulting solution and the cap was immediately shut to allow reaction with CO_2_ at 37 °C for 15 min. Released ^14^CO_2_ was adsorbed on the filter paper. After ice-cooling, the filter paper was put into a vial containing 3.5 mL of scintillation solution and radioactivity was counted three times for 4 min each. 

### 4.5. SAM and dcSAM Assay

Intracellular SAM and dcSAM concentrations were determined using reversed-phase HPLC. SAM and dc SAM were extracted from cells by the same method as that used for determination of the intracellular polyamine concentration. Using a Capcell pak C18 SG120 column (4.6 mm I.D. × 150 mm; Shiseido Co., Tokyo, Japan), 20 µL of sample was injected per assay for reversed-phase HPLC. The reversed-phase HPLC conditions were: column oven at 40 °C; flow rate, 0.5 mL/min; two solvent linear gradient, where solvent A consisted of 90% (*v*/*v*) 0.1 M sodium acetate (adjusted to pH 4.50 with acetate) with 10 mM sodium 1-octanesulfonate (Tokyo Chemical Industry Co., Tokyo) and 10% (v/v) methanol, and solvent B consisted of 90% (*v*/*v*) 0.2 M sodium acetate (pH 4.50)-acetonitrile (10:3) with 10 mM sodium 1-octanesulfonate and 10% (*v*/*v*) methanol; gradient, solvent B 0% to 100% over 50 min; UV detection at 250 nm; data were processed using LC Workstation chromatography software (Shimadzu Corp., Kyoto, Japan).

### 4.6. DNMT Assay and Activity Determination by Subtype

Since multiplication of HMEpCs was very slow, it was difficult to secure enough cells to measure protein levels and DNMT activities. Thus, these experiments were performed using Jurkat cells. An EpiQuik Nuclear Extraction Kit I (Epigentek Group, Farmingdale, New York (NY), USA) was used to extract nuclear proteins, using 1 μL of extraction buffer per 1.0 × 10^6^ cells. Protein concentrations of DNMT 1, 3A and 3B were determined using EpiQuik Assay Kits for each protein (all from Epigentek) (Epigentek Group Inc., NY, USA). After reacting nuclear protein solutions extracted from cells on plates covered with substances with high affinity for the respective DNMTs (37 °C for 2 h), the concentration of each DNMT was detected by a colorimetric method using a multiplate reader (DTX 880 Multimode Detector, Beckman Coulter Inc., Brea, California, USA) at 450 nm. 

DNMT 1, 3A and 3B activities were determined using an EpiQuik DNA Methyltransferase 1 Activity/Inhibitor Screening Assay Core Kit (Epigentek Group Inc., NY, USA), a DNMT3A Direct Activity Assay Kit (BPS Bioscience, San Diego, CA, USA), and an EpiQuik DNA Methyltransferase 3B Activity/Inhibitor Screening Assay Core Kit (Epigentek Group Inc., New York (NY), USA), respectively. Nuclear protein solutions were adjusted with assay buffer to make the protein concentrations of all DNMTs equivalent. Nuclear protein solutions were incubated with assay buffer on cytosine-rich DNA-covered plates (37 °C for 2 h). DNMT 1 and 3B were detected by a colorimetric method (450 nm) and DNMT 3A was detected by horseradish peroxidase chemiluminescence (1000 ms) using a multiplate reader. Analysis was conducted using Multimode Analysis Software ver. 3.2.0.5 (Beckman Coulter Inc. California, USA). DNMT activities are reported using indexes that were calculated by dividing measured activity by that of the control culture for each enzyme subtype.

### 4.7. Statistical Analysis

Statistical analyses were conducted using EZR software (Jichi Medical University Saitama Medical Center, Saitama-city, Japan) [60]. For analysis of differences between two groups, an unpaired *t*-test was used for homoscedasticity and the Mann Whitney test was used for heteroscedasticity. *p* < 0.05 was considered to indicate a significant difference in all analyses.

## 5. Conclusions

Decreases in ODC activity with aging is associated with decreases in DNMT activities and changes in DNA methylation status. Increased polyamine intake elevates blood spermine levels in mice and humans, and lifelong consumption of polyamine-rich chow inhibited aging-associated increase in aberrant DNA methylation, inhibited aging-associated pathological changes, and extend lifespan of mouse. The current study addresses the fundamental background of spermine-induced regulation of gene methylation leading to lifespan extension. In cells with decreased ODC activity, dcSAM/SAM ratio increased significantly. When ODC is suppressed, inhibition of AdoMetDC activity by spermine supplementation decreased dcSAM/SAM ratio. The decrease in dcSAM/SAM ratio is associated with an activation of DNMT 3a and 3b.

## Abbreviations

LFA-1	lymphocyte function-associated antigen 1
ODC	ornithine decarboxylase
SAM	S-adenosyl-L-methionine
DNMT	DNA methyltransferase
dcSAM	decarboxylated S-adenosylmethionine
AdoMetDC	S-adenosylmethionine decarboxylase
HMEpCs	human mammary epithelial cells
dcSAM	decarboxylated S-adenosylmethionine
